# Rapamycin's lifespan effect is modulated by mito‐nuclear epistasis in *Drosophila*

**DOI:** 10.1111/acel.14328

**Published:** 2024-09-03

**Authors:** Rita Ibrahim, Maria Bahilo Martinez, Adam J. Dobson

**Affiliations:** ^1^ School of Molecular Biosciences University of Glasgow Glasgow UK

**Keywords:** drosophila, genetic variation, Mito‐nuclear, quantitative genetics, rapamycin

## Abstract

The macrolide drug rapamycin is a benchmark anti‐ageing drug, which robustly extends lifespan of diverse organisms. For any health intervention, it is paramount to establish whether benefits are distributed equitably among individuals and populations, and ideally to match intervention to recipients' needs. However, how responses to rapamycin vary is surprisingly understudied. Here we investigate how among‐population variation in both mitochondrial and nuclear genetics shapes rapamycin's effects on lifespan. We show that epistatic “mito‐nuclear” interactions, between mitochondria and nuclei, modulate the response to rapamycin treatment. Differences manifest as differential demographic effects of rapamycin, with altered age‐specific mortality rate. However, a fitness cost of rapamycin early in life does not show a correlated response, suggesting that mito‐nuclear epistasis can decouple costs and benefits of treatment. These findings suggest that a deeper understanding of how variation in mitochondrial and nuclear genomes shapes physiology may facilitate tailoring of anti‐ageing therapy to individual need.

## Graphical abstract

The burden of ill health in older populations has motivated a search for anti‐ageing drugs, such as rapamycin, but are putative benefits equal among variable populations? Genes vary on DNA contained both in the nucleus and contained in mitochondria. In flies, this study demonstrates the principle that combined genetic variation in nuclei and mitochondria can interactively shape rapamycin's beneficial effects, by altering when in the lifespan the drug affects mortality.

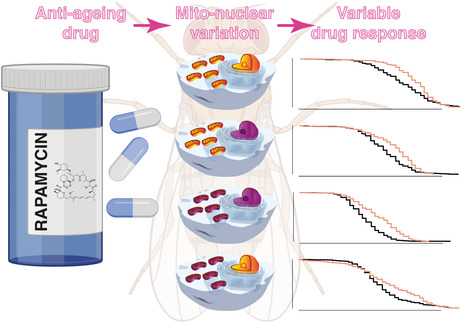

**Abbreviations**
AIC: Akaike Information Criterion
ANOVA: analysis of variance
EAA: essential amino acid
EMMeans: estimated marginal means
mtDNA: mitochondrial DNA
nDNA: nuclear DNA
SNP: single nucleotide polymorphism
TOR: Target of Rapamycin

## 1 INTRODUCTION

Ageing is characterised by loss of homeostasis and elevated disease risk (Bjedov & Partridge, 2011; Li et al., 2021; Partridge et al., 2018). To guard against age‐related pathology, there is considerable interest in drugging ageing itself. Rapamycin, an FDA‐approved anti‐inflammatory drug, robustly extends lifespan and healthspan in various animal models, leading to widespread interest in potential drug repurposing (Anisimov et al., 2011; Baroja‐Mazo et al., 2016; Bjedov & Rallis, 2020; Chen et al., 2009; Dai et al., 2014; Dumont & Su, 1996; Emran et al., 2014; Halloran et al., 2012; Harrison et al., 2009; Jia et al., 2004; Potter et al., 2019; Santiago et al., 2021). Responses to drugs can vary genetically (Heller, 2013; Madian et al., 2012; Roden et al., 2011; Scharfe et al., 2017), resulting in differential efficacy and safety, but surprisingly little attention has been paid to the potential for variable anti‐ageing effects of rapamycin.

Standing genetic variation in ageing (Clancy, 2008; Hall et al., 2019; Vaught et al., 2020) and genetically variable responses to anti‐ageing interventions other than rapamycin are already established, for example benefits of dietary restriction are genotype‐specific in mice (Liao et al., 2010, 2013; Swindell, 2012). Metformin, another drug‐repurposing candidate (Cabreiro et al., 2013), has variable effects on glycaemia (Florez, 2011; Nasykhova et al., 2022). But, to date, we are aware of just one study of variation in ageing responses to rapamycin, in *Drosophila* (Rohde et al., 2021). This phenotypic variation may be underpinned at least in part by mitochondrial DNA (mtDNA) variation, with rapamycin‐by‐mtDNA effects reported in metabolism and the transcriptome (Santiago et al., 2021; Villa‐Cuesta et al., 2014), but these studies did not address ageing. For any drug, understanding variable responses is vital, to ensure that benefits justify potentially costly side‐effects.

Why does genetic variation arise, and what is the potential role of mitochondria? Epistatic interactions among loci are thought to generate pervasive phenotypic variation (Boyle et al., 2017). Epistasis can occur between differently compartmentalised variation: In a phenomenon called mito‐nuclear epistasis, mtDNA variation modulates outcomes of nuclear DNA (nDNA) variation (Barshad et al., 2018; Mercer et al., 2011). This can affect mitochondrial function, for example only 13/80 proteins in the electron transport chain are mtDNA‐encoded, with the remainder encoded by nDNA, leading to potential “lock and key” effects on complex formation (Calvo & Mootha, 2010; Sunnucks et al., 2017). Reciprocally, outputs of mtDNA variation shape impacts of nDNA‐encoded variants, for example via bioenergetic changes that change penetrance of nDNA variants (Walker & Moraes, 2022). However, the role of mito‐nuclear variation in ageing is understudied. The knowledge gap extends to drugs: are responses to anti‐ageing drugs such as rapamycin subject to mito‐nuclear variation?

Here, we test whether mito‐nuclear epistasis shapes rapamycin's lifespan effect in *Drosophila*. This is timely because emerging evidence indicates that mito‐nuclear variation modulates translation (Barreto & Burton, 2013; Dobson et al., 2023; Hoekstra et al., 2013, 2018; Meiklejohn et al., 2013; Montooth et al., 2019), and translational regulation is thought to broker rapamycin's anti‐ageing effect (Filer et al., 2017). Further, we recently reported mito‐nuclear variation in the effects of dietary essential amino acids (EAAs) on egg laying in *Drosophila* (Dobson et al., 2023). EAAs are thought to promote egg laying via Target Of Rapamycin (TOR) signalling (Dobson et al., 2018; Emran et al., 2014), suggesting that TOR inhibition by rapamycin may also be subject to mito‐nuclear variation.

## 2 RESULTS

**FIGURE 1:**
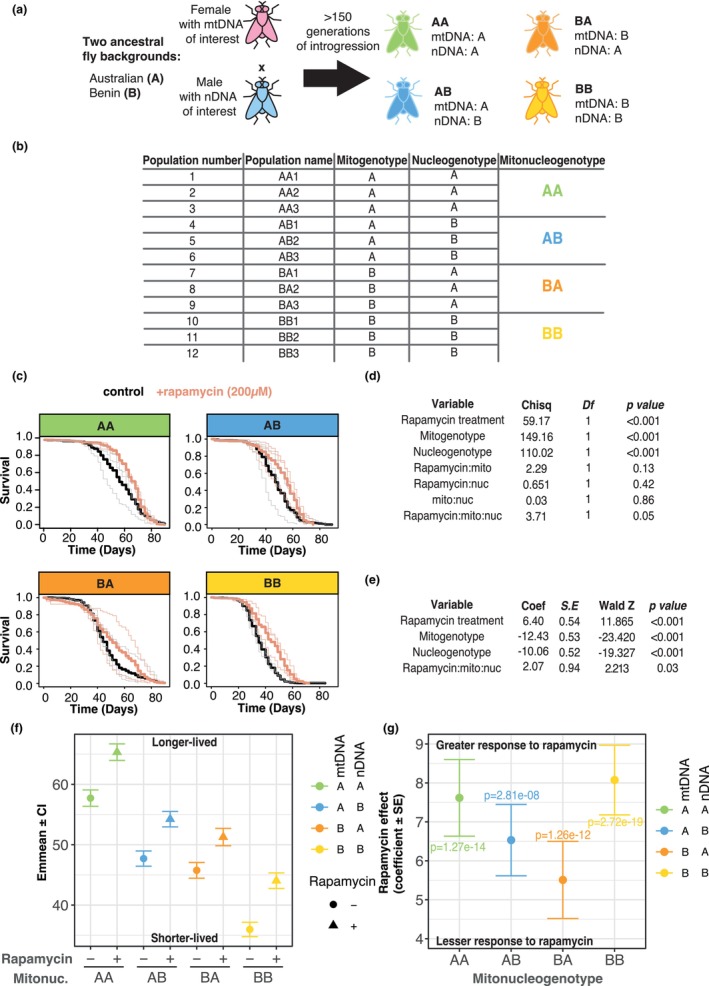
Rapamycin's effects on survival are modulated by mito‐nuclear epistasis. (a) Schematic showing how a panel of *Drosophila* with fully factorial mito‐nuclear combinations was generated from two ancestral fly backgrounds, isolated from New South Wales, Australia (*A*) and Benin (*B*). Females with the mitochondrial genome of interest were crossed iteratively to males with the nuclear genome of interest. This generated four mitonucleogenotypes, abbreviated to *AA, AB, BA* and *BB*. Three replicates were generated for each mitonucleogenotype, for 12 populations altogether. (b) The 12 independent populations, originating from two ancestral backgrounds A and B, were grouped to four mitonucleogenotypes based on their mitogenotypes and nucleogenotypes. (c) Kaplan–Meier survival plots showing the effect of lifelong rapamycin (200 μM) on survival of the four mitonucleogenotypes. Each facet shows a different mitonucleogenotype (3 replicate populations each), denoted along top. Heavy lines represent average survival across the three replicates (shown by light lines). (d) Parametric survival model (logistic distribution) of rapamycin‐mtDNA‐nDNA interactions (Type‐III ANOVA). (e) The output of a Fast Backward Variable Selection (fastbw) dropped the rapamycin:mito, rapamycin:nuclear and mito:nuclear two‐way interactions but retained the three‐way interaction. (f) Post hoc visualisation of differences among mitonucleogenotypes, using EMMeans from parametric survival model. (g) Coefficients for mitonucleogenotype‐specific responses to rapamycin (pairwise comparison of estimated marginal means) with standard errors, indicating that in mtDNA A background, nDNA variation does not modulate response to rapamycin, while in mtDNA B background, nDNA A has a lesser response to rapamycin than nDNA B. Coefficients are presented *‐1 so that higher values correspond to enhanced survival. For all plots, AA = mtDNA A and nDNA A; AB = mtDNA A and nDNA B; BA: mtDNA B and nDNA A; BB: mtDNA B and nDNA B.

mtDNA is inherited exclusively through eggs, while nDNA is inherited from both mothers and fathers. This enables us to manipulate mito‐nuclear variation by backcrossing a given nDNA (from fathers) to females bearing a given mtDNA, and then iterating the cross to enrich the paternal nDNA and purge the F0 mother's nDNA, while maintaining F0 mtDNA (Figure 1a). By this approach we generated *Drosophila* populations bearing fully‐factorial combinations of mtDNA and nDNA from Australia (A) and Benin (B). This resulted in four mitonucleogenotypes (AA = A mtDNA, A nuclei; AB = A mtDNA, B nDNA; BA = B mtDNA, A nDNA; BB=B mtDNA, B nDNA). For biological replication, three independent replicates of each mitonucleogenotype were produced in parallel (e.g., AA1, AA2, AA3, AB1, AB2, etc.). This produced 12 independent populations, comprising four mitonucleogenotypes (Figure 1b).

We derived our study populations from ancestral populations that were produced previously by hundreds of iterations of backcrossing (Dobson et al., 2023). DNA sequencing and SNP calling indicated that, as expected, this backcrossing procedure obliterated nDNA variation between replicate populations (i.e., AA indistinguishable from BA; and AB indistinguishable from BB). Backcrossing was relaxed for a period between the previous and present study (see materials and methods), which may have permitted some drift. We applied five iterations of backcrossing before commencing our study, which is expected to obliterate ~97% of nDNA differences that may have arisen by drift. We therefore expected negligible nDNA differences between populations sharing either A nDNA, or sharing B nDNA.

In contrast to nDNA, mtDNAs had potential to vary among replicates, because the ancestral replicate populations were derived from different F0 mothers which may have been segregating mtDNA variants. In our previous analysis of the ancestors of our current study populations (Dobson et al., 2023), we sequenced and identified mtDNA variants, finding greater variation between A vs B than among replicates of A or B: this suggests that the labels A or B are reasonable proxies for the majority of mtDNA variation in the study populations. However, since mtDNAs may also have drifted since our previous study, we conservatively assume that the previous mtDNA variant calls no longer apply. There remains a possibility for mtDNA variants to segregate between replicate populations, but we do not know which lines differ, nor at which mtDNA loci.

Altogether, the crossing design and our previous study of ancestors of the current populations led us to expect that A versus B nDNAs are differentiated, but the same nDNA across replicate populations should be negligibly different. We also expect mtDNAs A versus B to be well‐differentiated, but some variants may segregate among replicates as a founder effect of distinct F0 mothers.

Female flies from each of the 12 populations were fed 200 μM rapamycin from day three of adulthood until death (Bjedov et al., 2010), and survival was compared to vehicle‐fed controls (Figures 1c and S1, sample sizes in Table S1). We analysed the data with parametric survival models (logistic distribution). We first tested each of the 12 populations' responses to rapamycin independently (Figure S1), to assess whether differential responses to rapamycin grouped according to nDNA, or mtDNA, or potentially among replicate populations bearing co‐originating mtDNA (see above). Rapamycin did not shorten lifespan in any population. In 9 of the 12 populations, rapamycin extended lifespan (*p* < 0.05, Table S2), with a further marginal effect in one population (AA3 *p* = 0.07, Table S2). Rapamycin had no effect in the remaining two lines (BA2 *p* = 0.1, BB3 *p* = 0.19, Table S2). Thus, for each mitonucleogenotype, rapamycin extended lifespan in at least 2/3 independent replicate populations. Assuming that backcrossing successfully obliterated the majority of nDNA variation among replicates, uncharacterised variants among replicate mtDNAs are the most parsimonious explanation for the few among‐replicate differences we observed in response to rapamycin. However, since we did not have mtDNA sequence data, we took an approach consistent with the mito‐nuclear field and used population origin (i.e., A vs. B) of the mtDNAs as a surrogate for overall variation. This conservative approach averages across any potential mtDNA variation among replicates to reveal large‐scale trends.

Kaplan–Meier plots of survival per mitonucleogenotype (four pools of three replicate populations each) indicated genetically variable impacts of rapamycin (Figure 1c). A parametric survival model indicated a significant three‐way rapamycin*mtDNA*nDNA interaction (*p* = 0.05, statistical table in Figure 1d). The two‐way interactions (i.e., rapa:mito, rapa:nuclear, mito:nuclear) in this model were not significant (*p* > 0.05), and an automated model simplification algorithm (fastbw, Figure 1e) excluded these terms while retaining a significant rapa:mito:nuclear term (*p* = 0.03). This indicated that neither mtDNA nor nDNA alone were useful predictors of the variable response to rapamycin: rather, among‐line variation in response was driven by mito‐nuclear epistasis. To visualise this variation in response, we applied a post‐hoc estimated marginal means (EMMeans) analysis. Plotting EMMeans for each mitonucleoype ±rapamycin suggested differences among mitonucleogenotypes, which were modulated by rapamycin (Figure 1f). To visualise this directly, we extended the EMMeans analysis to estimate coefficients for impact of rapamycin in each mitonucleogenotype, with standard errors (Figure 1g). This indicated that, in mtDNA background A, switching from nDNA A to B did not affect response to rapamycin significantly (overlapping errors); but in mtDNA background B the same change of nDNA increased response to rapamycin (non‐overlapping errors). This indicated that the rapa‐mito‐nuclear effect in the study populations was due to a rapamycin‐nDNA interaction in mtDNA background B, which was absent in mtDNA background A.

**FIGURE 2:**
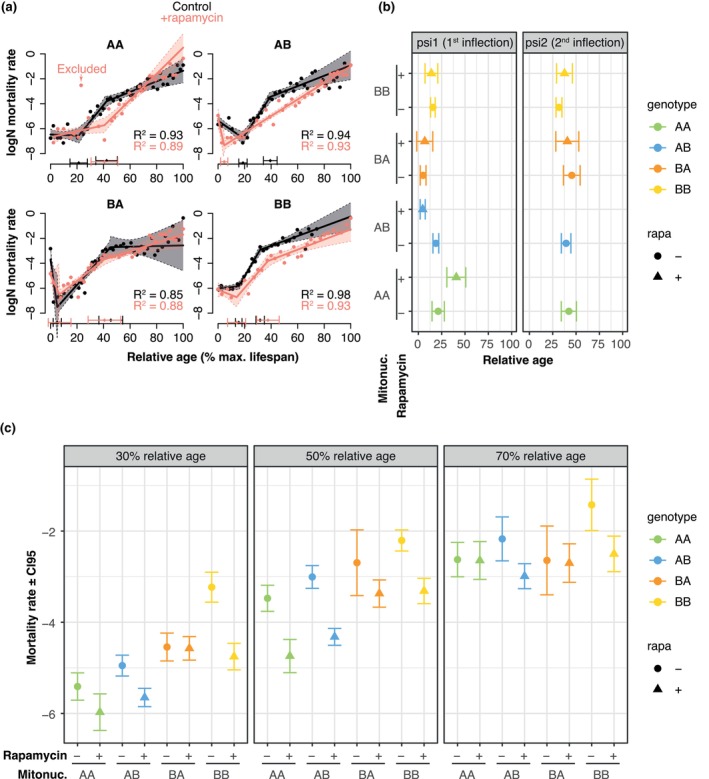
Mito‐nuclear epistasis modulates rapamycin's impact on mortality rate. (a) Panels show mortality rates (LogN) per mitonucleogenotype, across the lifecourse. Mitonucleogenotype is indicated above each panel. Model fits (weighted segmented regression) with 95% confidence intervals are indicated by lines. Time is shown as percentage of the total lifespan to mitigate confounding effects of differential maximum lifespan. Inflection points ± 95% confidence interval are shown above the *X*‐axis, and (b) Inflection points (psi) found by segmented regression of mortality rates over relative age. Points show estimate of inflection point ±95% confidence interval. A second inflection point is not given for conditions where the model with one inflection was preferred by AIC values. Non‐overlapping confidence intervals are taken to indicate significant differences in timing of inflection. (c) Time‐specific estimates of mortality rate. Time points for comparison were chosen to maximise equivalence of phases in the lifecourse. Mortality rates are taken to differ significantly when confidence intervals do not overlap. Note that the same values and confidence intervals are given as part of the continuous ribbon on facets in (a).

How did rapamycin‐mito‐nuclear variation manifest? The survival curves suggested that the interval in the lifecourse when rapamycin affected survival varied among mitonucleogenotypes, for example a late‐life survival difference in BB populations, contrasting an early‐to‐mid‐life difference in AA populations (Figure 1c). Plotting differences in Kaplan–Meier curves (i.e., rapamycin survival relative to controls) suggested that, indeed, survival impacts of rapamycin were altered by mito‐nuclear epistasis (Figure S2). To identify the demographic changes putatively underlying these survival differences, we calculated mortality rates, for all days on which deaths were observed. The mitonucleogenotypes varied in maximum lifespan, so to facilitate comparison between them on comparable scales we plotted mortality normalised to each mitonucleogenotype's maximum lifespan (Figure 2a; see Figure S3 for data presented without time normalisation). These plots indicated distinct patterns of mortality in different conditions, with inflection points in mortality rates that did not correspond between conditions. We therefore modelled the data using segmented regression—a method which identifies changes in slopes, and points on *X*‐variables when those changes occur. Segmented regression does not permit interaction terms, so we fit a regression to each mitonucleogenotype in the presence/absence of rapamycin, and compared predicted values with confidence intervals to identify significant differences between conditions. To mitigate against undue influence of datapoints at the end of the lifespan (Promislow et al., 1999), when mortality rates are estimated from small numbers of flies, we weighted the regressions by number of survivors. We excluded one datapoint from the model for AA flies on rapamycin, which appeared as an outlier and compromised model fit (datapoint highlighted in Figure 2a: AIC with point = 117.5, without point = 77.4. See Figure S3B for model with point included, and Figure S3C for diagnostic plots). We then fit a panel of segmented regressions allowing between zero to three inflection points, then selected the model with the optimal number of inflections based on AIC values. This yielded models explaining 85–98% of variance (R^2^ values given in Figure 2a).

Our approach allowed us to assess when inflections in trajectories occurred (Figure 2b), and compare mortality rates systematically among conditions (Figure 2c). We first examined when inflections occurred. Looking first at patterns in the absence of rapamycin, two inflections were evident in each mitonucleogenotype (Figure 2b). Early in life, mortality rates fell in AB and BA flies, but remained relatively constant in AA and BB flies. Mortality rates increased subsequently after an inflection point in all mitonucleogenotypes, before another inflection point at which increases slowed. Thus, in the absence of rapamycin, three distinct phases of mortality rate were apparent in all mitonucleogenotypes. Some mito‐nuclear variation was apparent in the transition from the first to the second phase (assessed by lack of overlap in confidence intervals), with inflection points in AA and AB flies not differing significantly, but the inflection was significantly earlier in BA than BB flies (non‐overlapping confidence intervals).

We then looked at how mortality rates changed over time after rapamycin feeding. Rapamycin changed the progression of mortality rate over time, but how it induced these changes depended on mito‐nuclear epistasis (Figure 2a). With B mitochondria, flies retained three phases of mortality rates, with a reduction in early life, an increase in early‐to‐mid life, then a lesser increase towards later life. By contrast, with A mitochondria, there were only two phases of mortality trajectory, with an early decrease followed by a monotonic increase after a single inflection point. However, this difference from other conditions appeared to be due to mito‐nuclear variation, because its timing depended on nDNA, appearing much earlier in AB flies than in AA flies. Thus, when our flies were fed rapamycin, mtDNA appeared to determine the number of phases of mortality trajectory, and mitonuclear epistasis determined when inflections in mortality rate occurred.

Having established when inflections occurred, we were then able to assess mortality rates quantitatively, in comparable phases of the lifespan. We chose to compare mortality rates at 30%, 50% and 70% through the lifespan, because these points did not coincide with inflections in mortality in any mitonucleogenotype. From the segmented regressions we calculated mortality rate ± CIs (Figure 2c). In the presence of nDNA B (i.e., AB and BB flies), rapamycin reduced mortality rate (non‐overlapping CIs) at all three sampling points, in the presence of either mtDNA A or B. By contrast, in BA flies, we detected no impact of rapamycin on mortality at any timepoint. However, the presence of mtDNA A (i.e., AA flies) restored rapamycin's effect, significantly reducing mortality rate at 50% lifespan (but not 30% or 50%). Thus, our results indicate that the demographic impact of rapamycin—when in the lifespan rapamycin will reduce mortality rate—is subject to mito‐nuclear epistasis. This may suggest that mito‐nuclear epistasis shapes which age‐dependent pathologies are alleviated by rapamycin, and perhaps the mechanisms by which mortality and survival are regulated.

**FIGURE 3:**
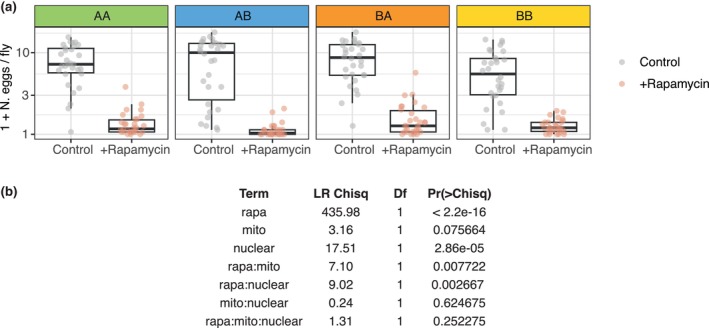
Reproductive cost of rapamycin is not modulated by mito‐nuclear epistasis. (a) Boxplots showing 24 h egg laying by 10‐day old females of the indicated mitonucleogenotypes, laid by 15 flies per vial (10 vials per condition), after 7 days feeding either on control or on rapamycin food. Mitonucleogenotypes are as described in Figure 1b. (b) Type‐3 ANOVA testing of negative binomial model of egg laying data. The absence of rapa‐mito‐nuclear epistasis indicates that reproductive costs of rapamycin are determined by processes independent of those that modulate ageing.

Our finding that mito‐nuclear epistasis impacts on when in the lifespan rapamycin impacts mortality relates to a broader question in the field—when is the optimal time to take the drug (Juricic et al., 2022)? Optimality is determined by the balance of costs and benefits, which led us to ask whether costs of rapamycin feeding would also be subject to mito‐nuclear effects. In flies, a major fitness cost of rapamycin feeding is reproductive arrest in youth (Emran et al., 2014). We therefore hypothesised that rapamycin's effect on reproduction, measured by egg laying, may also be subject to mito‐nuclear epistasis. We conducted a further experiment, quantifying egg laying over 24 h in 10 day‐old flies, after 7 days of rapamycin feeding (Figure 3). Rapamycin reduced egg laying in all four mitonucleogenotypes. While the magnitude of reduction varied, we did not detect a rapamycin‐mito‐nuclear interaction. Rather, we detected both a rapamycin‐nDNA interaction, and a rapamycin‐mtDNA interaction. These interactions contrasted directly with lifespan; suggesting that (A) fitness costs of rapamycin are not subject to mito‐nuclear epistasis, and (B) rapa‐mito‐nuclear epistasis for lifespan can in fact partially decouple lifespan benefits from fitness costs, which are subject to distinct axes of genetic variation. This suggests that (C) mito‐nuclear interactions, and lower‐order mitochondrial and nuclear interactions, have complex intersecting impacts on when it is optimal to take rapamycin.

## 3 DISCUSSION

To develop effective strategies to drug ageing, we need to understand the distribution of benefit among variable individuals and populations. Our data show that life‐long rapamycin treatment did indeed extend lifespan and reduce mortality among all mitonucleogenotypes under study, but the magnitude of effect varied. This was underpinned by mito‐nuclear variation, with effect of rapamycin treatment dependent on mtDNA in one nuclear background but not another. Mito‐nuclear epistasis is understudied in general but appears to explain significant phenotypic variance in *Drosophila*, and may be involved in human ageing (Dowling & Wolff, 2023; Tranah, 2011). These results build on studies that highlight mito‐nuclear effects on lifespan (Rand et al., 2006; Vaught et al., 2020; Zhu et al., 2014), and mtDNA effects on transcriptional responses to rapamycin (Santiago et al., 2021). The mechanisms underlying this variation remain to be elucidated, but we note evidence that mito‐nuclear interactions and rapamycin modulate similar cellular processes. For example, genetic evidence suggests that mito‐nuclear epistasis might modulate protein translation (Barreto & Burton, 2013; Dobson et al., 2023; Hoekstra et al., 2013, 2018; Meiklejohn et al., 2013; Montooth et al., 2019), although this is yet to be demonstrated biochemically. Rapamycin modulates translation as well, and some evidence suggests that this effect mediates its effect on lifespan (Bjedov et al., 2010; Filer et al., 2017; Martinez‐Miguel et al., 2021; Raught et al., 2001). Additional further mechanisms could involve the epigenome, as mitochondria are proposed to modulate health and ageing by altered epigenetic imprinting (Aon et al., 2016; Santos, 2021), and in flies rapamycin's anti‐ageing effects are also dependent on the epigenome (Lu et al., 2021). We propose that future investigation into the mechanisms of mito‐nuclear variation in lifespan should focus on translation and protein quality control, both specifically in mitochondria and in other organelles; and also epigenetics. We also propose that these mechanisms likely shape the response to rapamycin treatment.

To our knowledge, our data are the first evidence of mito‐nuclear variation in age‐specific mortality rate, and of an interaction with rapamycin. This might indicate that rapamycin alleviates different age‐related pathologies in different genotypes. This is an exciting prospect, as it may mean that drugs could be targeted precisely to alleviate specific pathologies experienced by specific individuals, if we can better understand the functional basis of mito‐nuclear epistasis. Much further work is now required to identify genotype‐specific changes in age‐dependent pathologies. A further important development will be to associate genetic variants, on both nDNA and mtDNA, with differential responses to rapamycin.

If age‐specific effects of an intervention vary genetically, might the optimum time to administer it co‐vary? In *Drosophila*, brief rapamycin treatment in early adulthood is reportedly sufficient for anti‐ageing effects, while late onset treatment was less beneficial (Juricic et al., 2022). Differences in response to timing and duration of rapamycin treatment urge future work to investigate whether these parameters interact with genetic variation, and whether demography is subsequently affected.

One caveat to our study is that, while nDNAs are expected to be homogenised among replicate populations by backcrossing, the crossing design (with each population originating from different F0 females) means that unidentified mtDNA variants may potentially persist among replicates. Such unidentified mtDNA variants are the most parsimonious explanation for the different responses to rapamycin that we observed in some replicate populations (Figure S1). Nevertheless, this work analyses geographic origin as a proxy for broad‐scale patterns of mtDNA variation, while controlling for among‐replicate nDNA variation. Future work in this space would benefit from controlled mtDNA haplotypes, for conclusive links to phenotype.

In conclusion, this study suggests that organismal impacts of rapamycin are genetically variable. Specifically, we demonstrate that epistasis between mtDNA and nDNA modulates response to rapamycin, and this alters patterns of age‐specific mortality. This has implications for development of methodologies to target anti‐ageing therapies precisely to the individual, which we suggest would be augmented by greater mechanistic understanding of how the interplay of genetic variation in mtDNA and nDNA modulates responses to rapamycin, and indeed other interventions. The ubiquity of variation in mtDNA and nDNA suggests that the phenomena we have modelled in flies will likely also be evident in other organisms.

## 4 EXPERIMENTAL PROCEDURES

### 4.1 Media

Flies were maintained on sugar‐yeast‐agar (1xSYA) medium containing 10% (w/v) brewer's yeast (MP Biomedicals SR03010, LOT No. S4707), 1.5% (w/v) agar (SIgma A7002), 5% (w/v) sucrose (Fisher BP220‐10) 3% nipagin, 0.3% propionic acid (Bass et al., 2007). Flies were cultured in a climate‐controlled insectary at 25°C on a 12:12 light/dark cycle, at 45–55% relative humidity. Rapamycin was purchased from LC Laboratories (R‐5000, LOT No. ASW‐149). Rapamycin stocked in ethanol (100 mM) was dissolved in fly medium to a final concentration of 200 μM. Ethanol was added to control media (2 mL per litre) as a vehicle control.

### 4.2 Fly stocks and husbandry

We re‐established a panel of *Drosophila melanogaster* populations from preceding populations, supplied by Professor Klaus Reinhardt, Technische Universität Dresden, Germany (Dowling & Wolff, 2023). These populations had nDNA introgressed into mtDNA backgrounds over hundreds of generations. We chose to use these lines because variation in fitness effects of diet—which is expected to intersect with TOR signalling—has been reported previously (Dobson et al., 2023). After we took receipt of these populations, flies were maintained without backcrossing for ~6 months during the 2020 coronavirus outbreak. It is therefore likely that drift occurred while introgression was relaxed and, therefore, we conservatively discount mtDNA genotype calls presented previously (Dobson et al., 2023). Instead, we re‐established new populations from the descendants of the preceding populations, retaining mtDNAs by taking virgin mothers (*n* = 45 per populations), and then backcrossing males (n = 45 per population) from the original Australian or Benin stock populations for five generations (as per (Dobson et al., 2023)) (Figure 1a). mtDNAs and nDNAs were backcrossed in this way to re‐establish fully‐factorial combinations of mtDNAs and nDNAs, with three replicates per populations (Figure 1b). Our previous analysis (Dobson et al., 2023) suggested that this design should homogenise nDNAs across replicate populations, irrespective of mtDNA; however subtle but unavoidable mtDNA variations can potentially arise.

### 4.3 Lifespan assay

For each population, egg‐collection cages with males and females were set up with clementine juice plates (In 1 L of clementine juice agar: 20 g agar, 26 g sucrose, 52 g glucose, 7 g yeast, 88.8 mL organic clementine juice, 800 mL distilled water). Eggs were collected from the plates, suspended in PBS, and a 20 μL suspension was pipetted into 60 mL of media in a 170 mL fly bottle. Ten days later, newly‐eclosed flies were transferred to fresh bottles and were sorted after 48 h, under CO_2_ anaesthesia. Mated female flies were split into groups of 15, and transferred into 10 vials per condition (rapamycin and control). Males were discarded. The flies were transferred to fresh food and deaths were scored three times per week.

### 4.4 Egg laying assay

Flies were reared to adult, mated, and then fed rapamycin as above. After 7 days of rapamycin feeding, egg deposition over 24 h on fresh media was quantified by discarding flies, freezing vials containing eggs and media at −20°C, and subsequently counting eggs with a stereoscope.

### 4.5 Statistical analysis

The lifespan and fecundity data were analysed in R 4.3.2. Data and code have also been uploaded to github.com/dobdobby.

Survival data were analysed with the *rms* library, by fitting a parametric survival model (rms::psm) with a logistic distribution, of the form:

Survival~rapamycin×mtDNA×nDNA

The model with the logistic distribution was selected on the basis of AIC by comparison against models of the same form using Weibull, exponential, Gaussian, logNormal and log‐logistic distributions, each of which had greater AIC values. Fast Backward Variable Selection (fastbw) was performed (rms::fastbw) for model simplification. Differences between Kaplan–Meier curves for rapamycin‐fed and control animals were plotted with rms::survdiffplot. Estimated marginal means and effects of rapamycin were calculated using the *emmeans* library with emmeans::emmeans and emmeans::pairs, respectively. Per‐line effects of rapamycin (Table S2) were calculated with emmeans::joint_tests. P‐values from these tests are corrected for multiple hypothesis testing (Tukey). Type‐3 ANOVA tests were applied using the Car library (Car::Anova), with contrasts set to “contr.sum”.

Mortality rate estimates were calculated in Microsoft Excel, following formulae from (Promislow et al., 1999). At timepoints (*x*) when deaths were observed, we calculated proportion surviving (*l*_*x*_). We then calculated an estimate of age‐specific mortality rate:

$$q_{x}={\mathrm{\Delta l}}_{x}/\left(l_{x}\times \mathrm{\Delta x}\right)$$

from which we calculated an estimate of instantaneous mortality rate

$$\mu_{x}=-\text{logN}\left(1-q_{x}\right)$$

To enable comparisons among mitonucleogenotypes with different maximum lifespans, we normalised x as a percentage of each population's maximum lifespan (i.e., longest‐lived individual). *μ*_*x*_ was then modelled using linear regression (lm) and segmented regressions with the *segmented* R package, by first fitting a linear model, and then segmented regressions with segmented::segmented. For each of the eight starting linear models (i.e., four mitonucleogenotypes × two drug conditions), we fit three segmented regressions where ψ (i.e., N. inflections of y/x) equalled 1, 2 or 3. We then selected the best model from AIC values. All models were weighted by number of survivors to mitigate against effects of estimating *μ*_*x*_ from small numbers of survivors towards the end of the experiment. Inflection points and confidence intervals were extracted from the segmented regressions with segmented::confint, and mortality rates with 95% confidence intervals were calculated both throughout the lifespan (Figure 2a) and specifically at 30%, 50% and 70% through the lifespan (Figure 2c) using the stats::predict function.

One datapoint was excluded from the segmented regression for AA flies fed rapamycin. The point is highlighted in main figures (Figure 2a), and the fit with this point included is shown in Figure S3B. Diagnostic plots identified this point specifically as an outlier (Figure S3C), and its exclusion improved AIC values (from 117.5 to 77.4).

Fecundity data were analysed by dividing number of eggs per vial by number of females in the vial (15 in all cases), and modelling the data with a negative binomial model fitted with the MASS library (MASS::glm.nb) of the form

N.eggs~rapamycin×mtDNA×nDNA.

Type‐3 ANOVA tests were applied using the Car library (Car::Anova), with contrasts set to “contr.sum”.

## AUTHOR CONTRIBUTIONS

RI and AJD conceived the study. RI and MBM acquired data. RI and AJD performed data analysis. AJD supervised the study. RI and AJD wrote the manuscript.

## CONFLICT OF INTEREST STATEMENT

The authors declare no conflicts of interest.

## Supporting information


**Figure S1.** Impacts of rapamycin on each replicate population within the three mitonucleogenotypes. Data are as per Kaplan–Meier graphs in Figure 1, plotted with each replicate population on its own pair of axes. In Figure 1 the same data are shown (as light lines) along with average of three replicates (as heavy lines).

**Figure S2.** Effects of rapamycin on Kaplan–Meier plots appear to vary at distinct points in the lifespan. Panels show survdiffplots for the indicated mitonucleotypes, where the line at zero represents control conditions and the variable line represents the difference (± confidence interval) in rapamycin‐fed flies. Graphs are overlaid to show how mitonucleogenotype appears to alter when in the lifespan rapamycin impacts survival. (A) All mitonucleogenotypes, and (B) pairwise comparisons.

**Figure S3.** Mortality rate analysis. (A) Mortality rate analysis as per Figure 2a, without normalisation of time over maximum lifespan. (B) An outlier was excluded from the segmented regression analysis of AA flies after rapamycin feeding (indicated on Figure 2a,S3A). The regression is shown here after inclusion of the outlier. (C) Diagnostic plots of the model shown in B consistently identify one datapoint (7) as an outlier. The point was therefore excluded from the analysis presented in Figure 2a.

Table S1.

## Data Availability

The data that support the findings of this study are openly available at github.com/dobdobby/rapa‐mito‐nuclear.
