# Supplementary figures and images for: Rapamycin's lifespan effect is modulated by mito‐nuclear epistasis in Drosophila
Source: Aging Cell. 2024 Sep 3;23(12):e14328. doi: 10.1111/acel.14328 (PMC11634709; doi:10.1111/acel.14328)

Figure S1

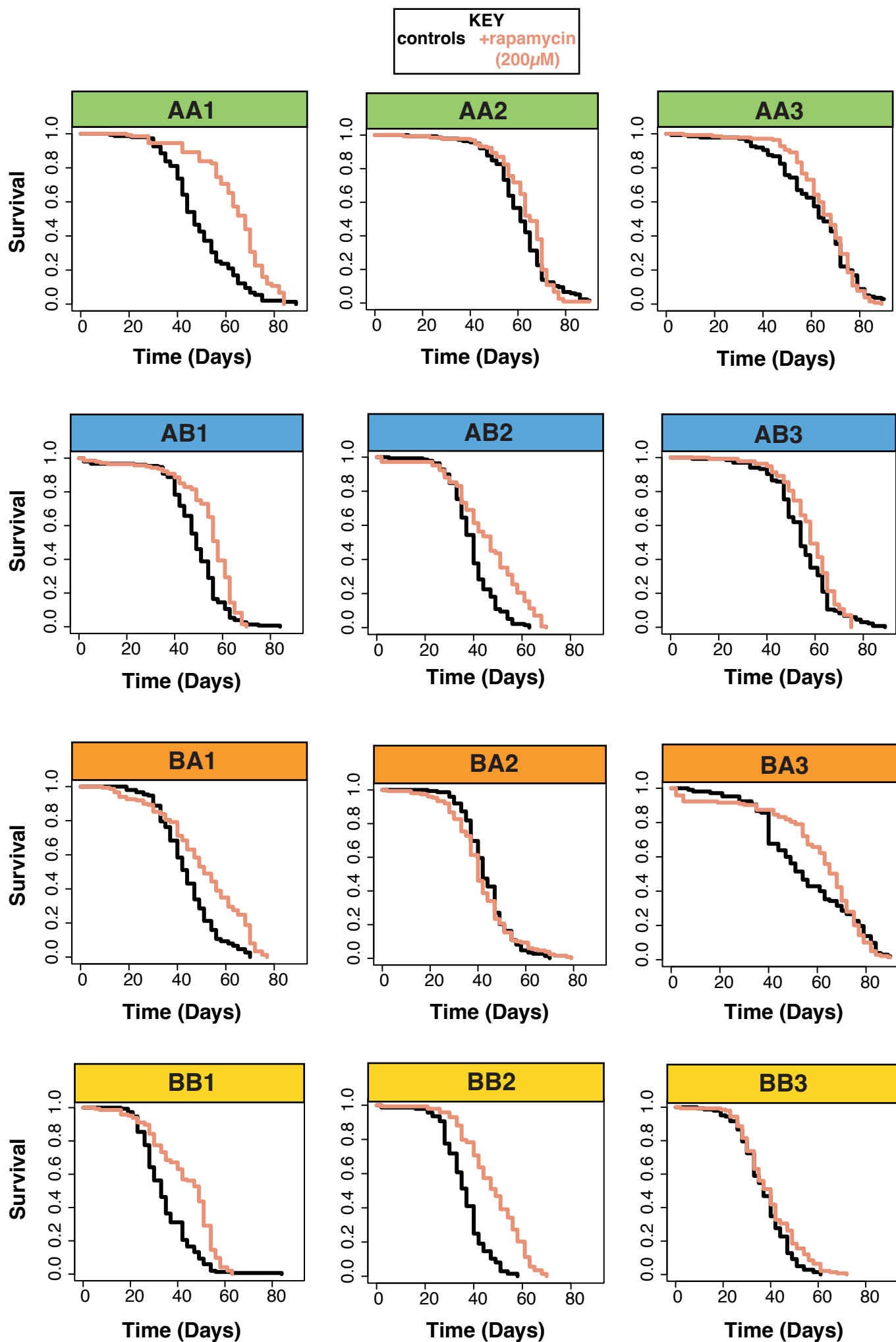

Supplement: Supplementary file 1 — Figure S1. Impacts of rapamycin on each replicate population within the three mitonucleogenotypes. Data are as per Kaplan–Meier graphs in Figure 1, plotted with each replicate population on its own pair of axes. In Figure 1 the same data are shown (as light lines) along with average of three replicates (as heavy lines). [file ACEL-23-e14328-s002.pdf]

Figure S2

A

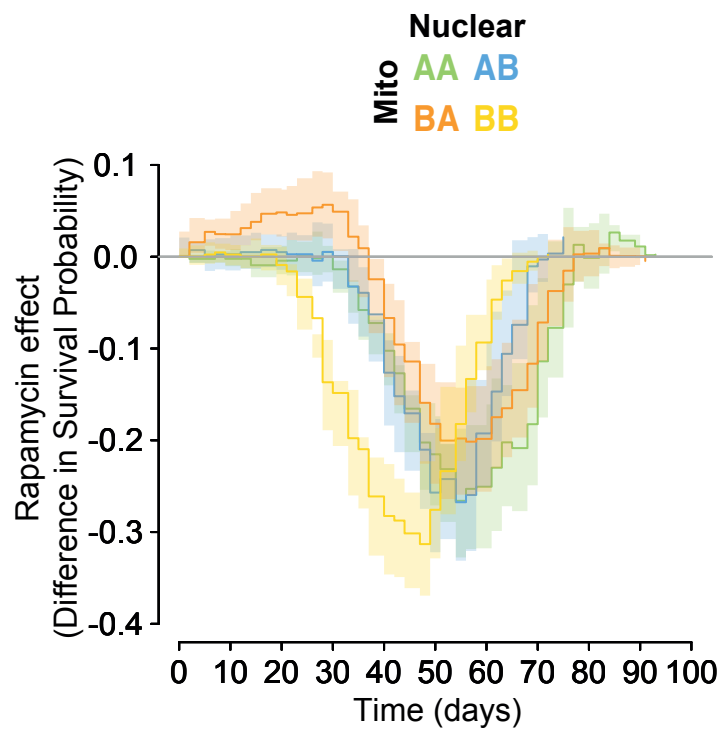

B

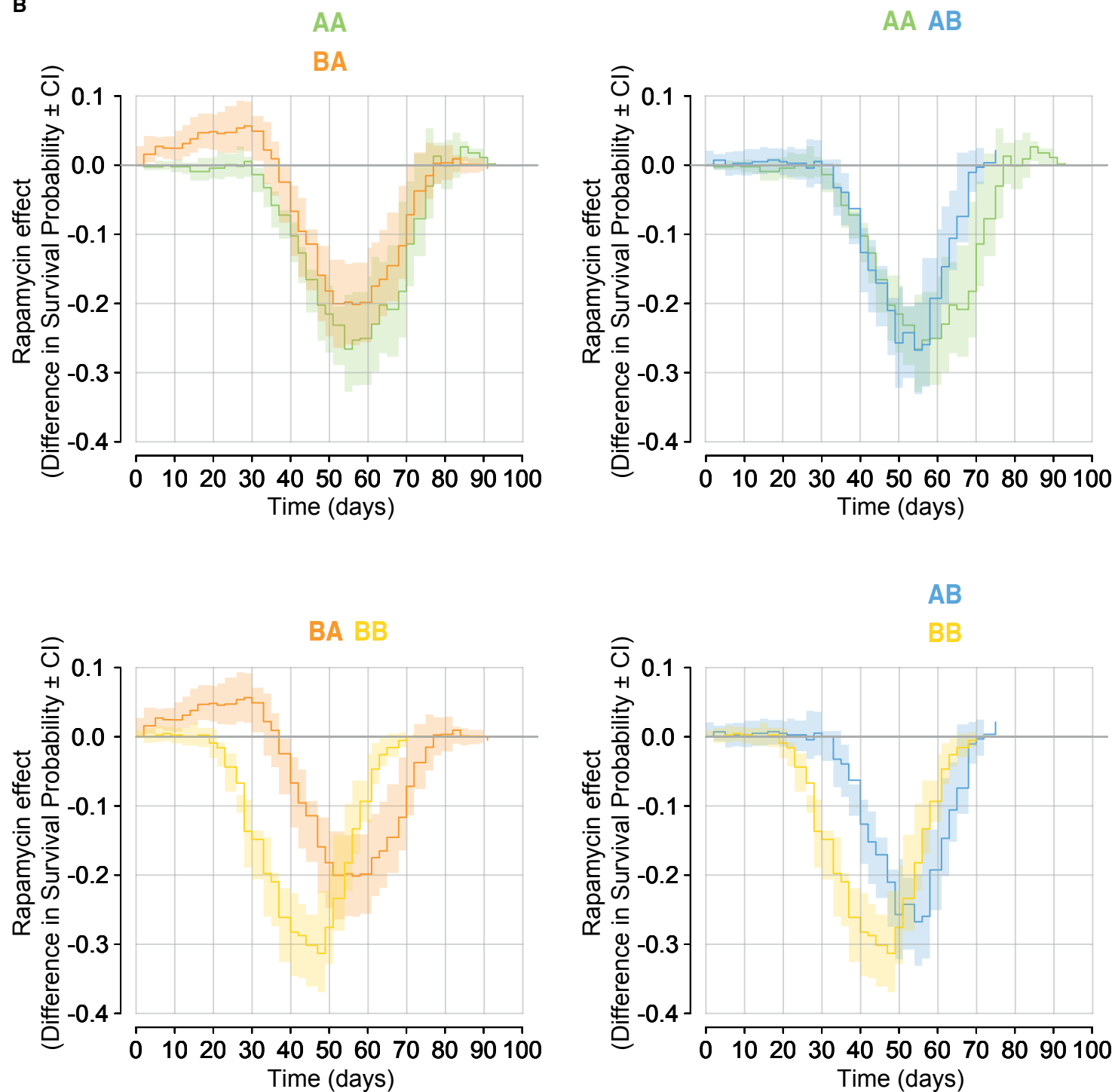

Supplement: Supplementary file 2 — Figure S2. Effects of rapamycin on Kaplan–Meier plots appear to vary at distinct points in the lifespan. Panels show survdiffplots for the indicated mitonucleotypes, where the line at zero represents control conditions and the variable line represents the difference (± confidence interval) in rapamycin‐fed flies. Graphs are overlaid to show how mitonucleogenotype appears to alter when in the lifespan rapamycin impacts survival. (A) All mitonucleogenotypes, and (B) pairwise comparisons. [file ACEL-23-e14328-s003.pdf]

Figure S3

A

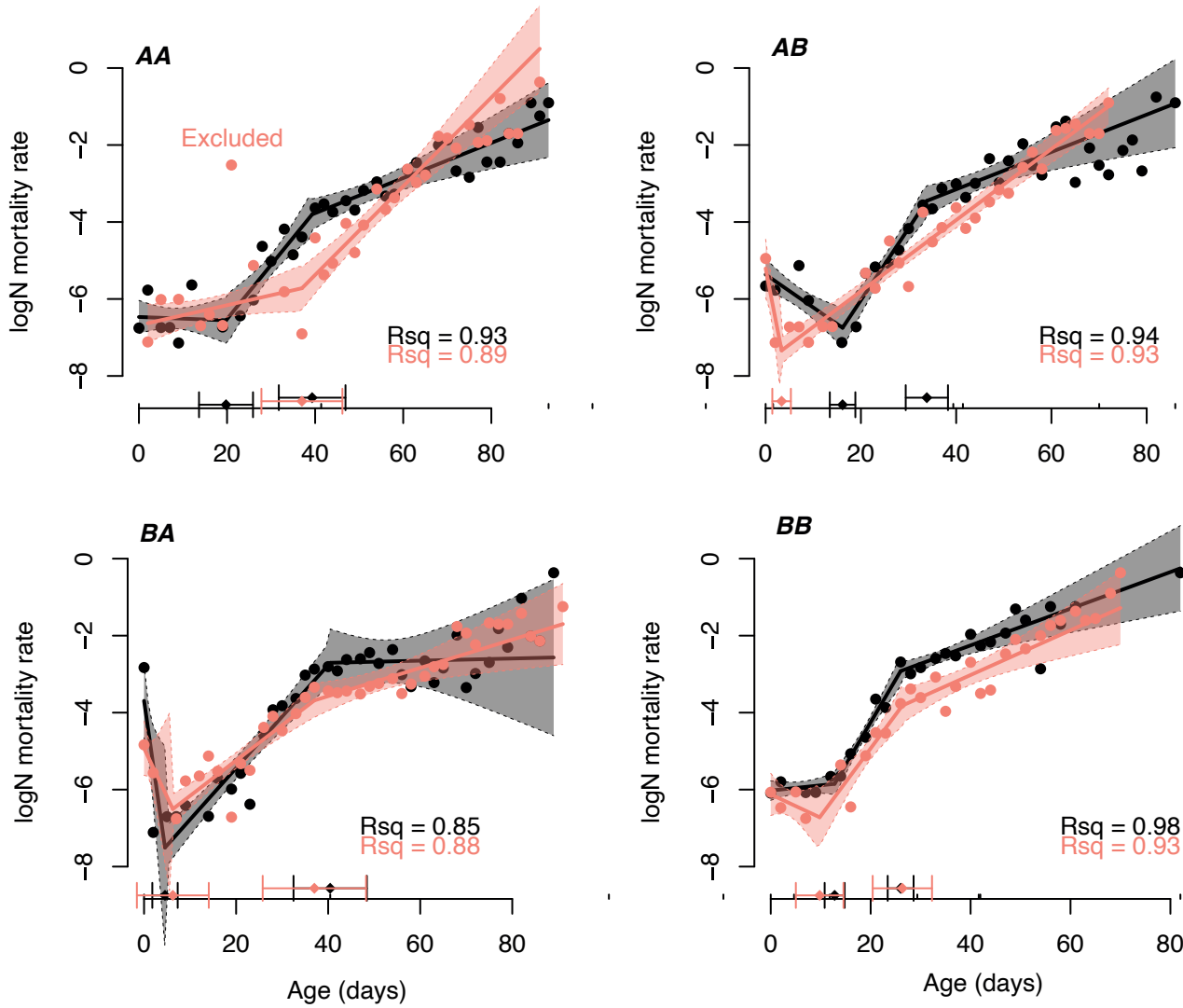

B

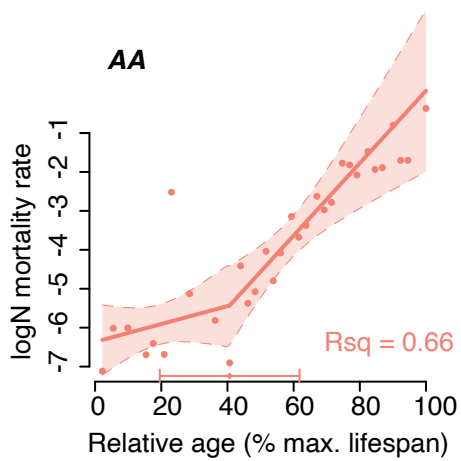

C

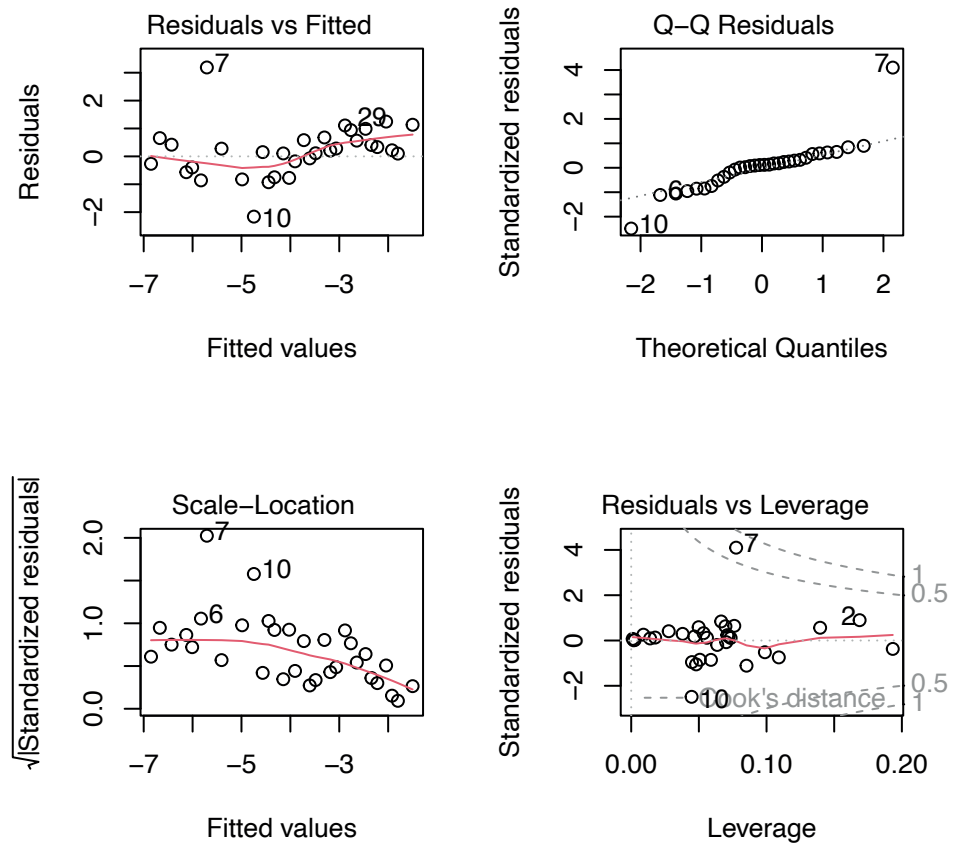

Supplement: Supplementary file 3 — Figure S3. Mortality rate analysis. (A) Mortality rate analysis as per Figure 2a, without normalisation of time over maximum lifespan. (B) An outlier was excluded from the segmented regression analysis of AA flies after rapamycin feeding (indicated on Figure 2a,S3A). The regression is shown here after inclusion of the outlier. (C) Diagnostic plots of the model shown in B consistently identify one datapoint (7) as an outlier. The point was therefore excluded from the analysis presented in Figure 2a. [file ACEL-23-e14328-s004.pdf]
