# Supplementary material for: Rapamycin's lifespan effect is modulated by mito‐nuclear epistasis in Drosophila
Source: Aging Cell. 2024 Sep 3;23(12):e14328. doi: 10.1111/acel.14328 (PMC11634709; doi:10.1111/acel.14328)
Supplement: Supplementary file 4 — Table S1. [file ACEL-23-e14328-s001.docx]

| **Table S1. Numbers of flies per population and rapamycin treatment used in the ageing study.** | | |
| --- | --- | --- |
| **Population** | **- rapamycin** | **+ rapamycin** |
| AA1 | 149 | 137 |
| AA2 | 139 | 136 |
| AA3 | 141 | 138 |
| AB1 | 152 | 142 |
| AB2 | 145 | 143 |
| AB3 | 135 | 139 |
| BA1 | 152 | 149 |
| BA2 | 152 | 151 |
| BA3 | 158 | 143 |
| BB1 | 154 | 147 |
| BB2 | 142 | 143 |
| BB3 | 146 | 143 |

| **Table S2. Effect of rapamycin per population. Parametric survival model for population * rapamycin, stratified by *post hoc* tests (Emmeans - joint tests) per population.** | | | | | |
| --- | --- | --- | --- | --- | --- |
| **term** | **line** | **df1** | **df2** | **F ratio** | **p value** |
| rapa | AA1 | 1 | 3451 | 90.693 | 3.05E-21 |
| rapa | AA2 | 1 | 3451 | 4.214 | 0.04016526 |
| rapa | AA3 | 1 | 3451 | 3.355 | 0.06707599 |
| rapa | AB1 | 1 | 3451 | 26.352 | 3.00E-07 |
| rapa | AB2 | 1 | 3451 | 23.623 | 1.22E-06 |
| rapa | AB3 | 1 | 3451 | 6.84 | 0.00895239 |
| rapa | BA1 | 1 | 3451 | 28.451 | 1.02E-07 |
| rapa | BA2 | 1 | 3451 | 2.686 | 0.10133871 |
| rapa | BA3 | 1 | 3451 | 25.985 | 3.63E-07 |
| rapa | BB1 | 1 | 3451 | 54.636 | 1.81E-13 |
| rapa | BB2 | 1 | 3451 | 71.1 | 4.92E-17 |
| rapa | BB3 | 1 | 3451 | 1.743 | 0.18685447 |
